# Repeated exposure to systemic inflammation and risk of new depressive symptoms among older adults

**DOI:** 10.1038/tp.2017.155

**Published:** 2017-08-15

**Authors:** J A Bell, M Kivimäki, E T Bullmore, A Steptoe, MRC ImmunoPsychiatry Consortium, Edward Bullmore, Petra E Vértes, Rudolf Cardinal, Sylvia Richardson, Gwenael Leday, Tom Freeman, David Hume, Tim Regan, Zhaozong Wu, Carmine Pariante, Annamaria Cattaneo, Patricia Zuszain, Alessandra Borsini, Robert Stewart, David Chandran, Livia A Carvalho, Joshua A Bell, Luis Henrique Souza-Teodoro, Hugh Perry, Neil Harrison, Wayne Drevets, Gayle M Wittenberg, Yu Sun, Declan Jones, Edward Bullmore, Shahid Khan, Annie Stylianou, Robert B Henderson, L A Carvalho

**Affiliations:** 1 Department of Epidemiology & Public Health, University College London, London, UK; 2 Department of Psychiatry, University of Cambridge, Cambridge, UK; 3 Department of Clinical Pharmacology, William Harvey Research Institute, Charterhouse Square, Barts and The London School of Medicine and Dentistry, Queen Mary University of London, London, UK

## Abstract

Evidence on systemic inflammation as a risk factor for future depression is inconsistent, possibly due to a lack of regard for persistency of exposure. We examined whether being inflamed on multiple occasions increases risk of new depressive symptoms using prospective data from a population-based sample of adults aged 50 years or older (the English Longitudinal Study of Ageing). Participants with less than four of eight depressive symptoms in 2004/05 and 2008/09 based on the Eight-item Centre for Epidemiologic Studies Depression scale were analysed. The number of occasions with C-reactive protein ⩾3 mg l^−1^ over the same initial assessments (1 vs 0 occasion, and 2 vs 0 occasions) was examined in relation to change in depressive symptoms between 2008/09 and 2012/13 and odds of developing depressive symptomology (having more than or equal to four of eight symptoms) in 2012/13. In multivariable-adjusted regression models (*n*=2068), participants who were inflamed on 1 vs 0 occasion showed no increase in depressive symptoms nor raised odds of developing depressive symptomology; those inflamed on 2 vs 0 occasions showed a 0.10 (95% confidence intervals (CIs)=−0.07, 0.28) symptom increase and 1.60 (95% CI=1.00, 2.55) times higher odds. In further analyses, 2 vs 0 occasions of inflammation were associated with increased odds of developing depressive symptoms among women (odds ratio (OR)=2.75, 95% CI=1.53, 4.95), but not among men (OR=0.70, 95% CI=0.29, 1.68); *P*-for-sex interaction=0.035. In this cohort study of older adults, repeated but not transient exposure to systemic inflammation was associated with increased risk of future depressive symptoms among women; this subgroup finding requires confirmation of validity.

## Introduction

Mental disorders contribute greatly to the global burden of disease^1^ and depression is the largest driver of morbidity associated with these disorders.^2^ A large body of evidence supports immune dysfunction in the form of low-grade systemic inflammation as a characteristic of depression, with meta-analyses having established higher circulating levels of pro-inflammatory factors C-reactive protein (CRP), interleukin-6 (IL-6) and tumour necrosis factor alpha (TNF-α) among adults with than without depressive disorders.^3^ Still unclear, however, is whether systemic inflammation is a risk factor for future depression.

Plausible pathways exist for depressogenic effects of inflammation including disrupted metabolism of mood-enhancing neurotransmitters, creation of neurotoxic metabolites and chronically heightened stress responses,^4, 5, 6^ but prospective population-based findings are mixed^7^ with reports of both a positive link^8, 9, 10, 11^ and of no such link.^12, 13, 14^ One possible explanation for these mixed results is the issue of inflammation chronicity. It is reasonable to expect that pro-inflammatory signals need to persist over time in order to induce depression; however, most studies conducted thus far have measured inflammation only once and cannot capture these potential effects. This leaves unanswered the question of whether exposure to systemic inflammation is associated with risk of developing depression in a dose–response manner; an observation that would support an argument for causality.

Depression is also known to be more common among women than men.^15^ Miller and Raison^16^ have theorised that this may partly reflect an evolutionary adaptation among women such that depressive symptoms function as avoidance behaviour with the effect of minimising infection by external pathogens in reproductive years.^17^ Studies have suggested that women react more strongly than men to stressful stimuli by way of heightened inflammation,^18^ and are more likely than men to develop depressive symptoms in response to equal amounts of drug-induced pro-inflammatory cytokines.^19^ It may therefore be expected that women are most affected by repeated exposure to systemic inflammation by way of greater depression risk; studies examining this are scarce.

We examined whether repeated exposure to systemic inflammation increases the risk of developing new depressive symptoms by analysing repeated measures of CRP from a population-based sample of older adults.

## Materials and methods

### Study population

Data were drawn from the English Longitudinal Study of Ageing (ELSA), a nationally representative panel study of community-dwelling adults aged 50 years and older in England.^20^ Questionnaire data have been collected every 2 years since 2002/03 and biological data have been collected every 4 years since 2004/05. Participants gave informed written consent to participate and ethical approval was obtained from the London Multi-centre Research Ethics Committee.

### Assessment of systemic inflammation

High-sensitivity CRP was used as a blood-based marker of systemic inflammation, measured in serum using the N Latex high-sensitivity CRP mono immunoassay on the Behring Nephelometer II analyser with a 0.17 mg l^−1^ detection limit and a coefficient of variation <6%.^21^ Measurements of CRP from two occasions (2004/05 and 2008/09) were used for this study. Participants were considered to be inflamed on each occasion if their CRP was 3 mg l^−1^ or higher based on recommendations for cardiovascular outcomes.^22^ We note that an equivalent cut-point is not available for mental health outcomes and that 3 mg l^−1^ corresponds approximately to the upper tertile of CRP values in our sample.

### Assessment of depressive symptoms

Depressive symptoms were assessed in a consistent manner on three occasions, once in 2004/05, once in 2008/09 and once in 2012/13 using symptoms identified through the Centre for Epidemiologic Studies Depression (CES-D short version) eight-item scale, which has been well validated in samples of older adults.^23, 24, 25^ Items referred to experiences in the past week and included feelings of depression, sadness, loneliness, a lack of enjoyment, a lack of happiness, that everything was an effort, that they ‘could not get going’ and that their sleep was restless. The CES-D score represented the total number of depressive symptoms (out of 8) that a participant reported having. Scoring positive for at least four of the eight symptoms questioned in the short version CES-D is often considered to represent a threshold for the binary presence of depressive symptomology,^26, 27, 28^ which is similar to the threshold of 16 used in the longer version CES-D_20_.

### Assessment of covariates

Data on measured covariates were used from 2008/09 to coincide with the main study baseline. At both time points, demographic factors included participant age, sex and wealth. Wealth summarised participants’ accumulated resources based not on pension income but on the debt-free value of major assets (that is, housing, investments and goods).^20^ Measured height (in metres, m) and weight (in kilograms, kg) were used to calculate body mass index (BMI, in kg/m^2^). Chronic disease burden was examined through self-reported physician diagnosis of a range of diseases that are common at older ages including cardiovascular disease (myocardial infarction, angina or stroke), type 2 diabetes, cancer, osteoarthritis, rheumatoid arthritis, chronic lung disease and asthma. Disability was assessed using 14 items on basic and instrumental activities of daily living that cover daily tasks required for independent functioning including the ability to prepare food, to bathe and to make telephone calls; participants were considered disabled if they reported ⩾1 such limitation. Cognitive impairment was indicated by poor performance on two cognitive tests, first being a memory test based on the total number of words recalled immediately and after a delay from a list of 10 words (each presented 2 s apart), and the second being an executive function test based on the total number of exemplars of a given category (animals) named in 1 min. Such measures have been validated against clinical assessments of neuropathology during autopsy^29^ and extensively used in ELSA^30, 31, 32^ and other epidemiological studies. For present analyses, scores on each test were standardised and summed, and participants scoring 1 s.d. below the sample mean on this summed score were considered cognitively impaired. Participants’ smoking status was categorised as never-smoker, former smoker or current smoker. Participants’ self-reported frequency of physical activity was divided into three groups based on doing moderate or vigorous activities: (1) more than once/week, (2) once/week or (3) one to three times/month, hardly ever or never. Use of antidepressant medication was identified through British National Formulary codes for tricyclic antidepressants (4.3.1), selective serotonin reuptake inhibitor antidepressants (4.3.3) and other antidepressants (4.3.4), as recorded by a nurse in 2012/13.

### Statistical approach

**Figure 1:**
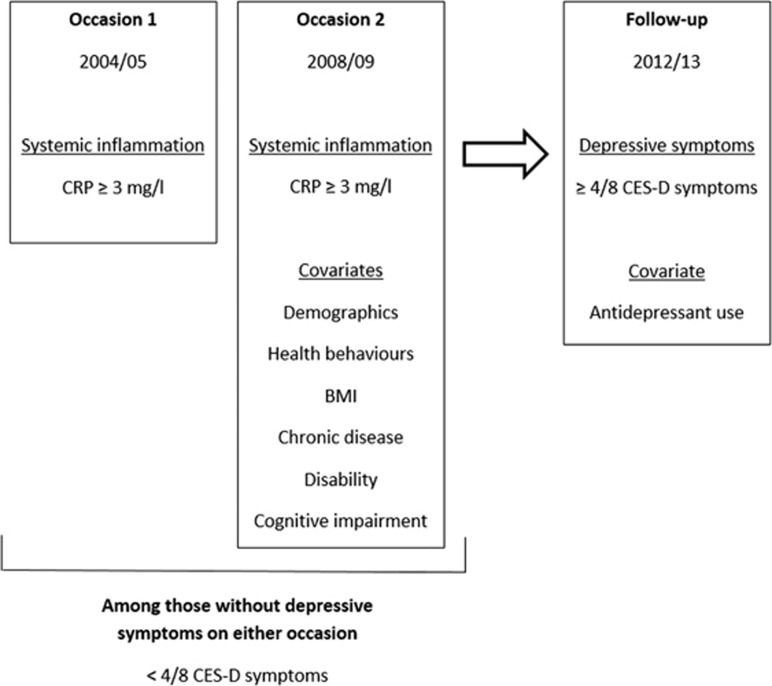
Outline of the prospective study design for developing depressive symptoms using the English Longitudinal Study of Ageing. BMI, body mass index; CES-D, Centre for Epidemiologic Studies Depression; CRP, C-reactive protein.

All analyses were conducted on participants who were free of depressive symptoms (had less than four of eight symptoms) in both 2004/05 and 2008/09 (Figure 1).

We first examined the number of occasions with high CRP (1 vs 0, and 2 vs 0) in relation to continuous change in the number of depressive symptoms from the main study baseline until the end of follow-up by subtracting the 2008/09 symptom score value from the 2012/13 symptom score value, using linear regression. The first model adjusted for age, sex and wealth. Adjustments were made for measured covariates in a series of additional models: the second included antidepressant drug use in 2012/13 (coinciding with follow-up depressive symptom assessment); the third included BMI, each individual chronic disease, disability and cognitive impairment; the fourth included smoking and physical activity; and the fifth included all factors collectively.

Logistic regression models were then used to compare the odds of having depressive symptoms (more than or equal to four of eight symptoms) in 2012/13 according to the number of occasions (1 vs 0, and 2 vs 0) with high CRP, measured in 2004/05 and 2008/09 (the two occasions that preceded the follow-up assessment of depressive symptoms). Analyses were repeated on a subset of participants who were free of all eight depressive symptoms in 2004/05 and 2008/09 in order to compare the odds of developing each individual symptom in 2012/13 according to the number of occasions inflamed.

For each analysis, an interaction was tested between participant sex and the exposure (number of occasions inflamed) in relation to the outcome (continuous change in the number of depressive symptoms or binary development of depressive symptomology), adjusting for age, sex and wealth.

### Sensitivity analyses

Given the possibility that very high values of CRP may represent an occult inflammatory disorder, analyses of repeated inflammation in relation to continuous change in the number of depressive symptoms and binary development of depressive symptomology were repeated on a subset of participants who had CRP values <10 mg l^−1^ on each occasion, as recommended for cardiovascular disease prevention,^22^ again due to lack of a recognised cut-point for depressive outcomes.

All analyses were performed using SPSS version 24 (SPSS, Chicago, IL, USA), with two-tailed *P*<0.05 guiding statistical significance.

## Results

### Included and excluded study populations

A total of 2068 participants were free of depressive symptoms (less than four of eight symptoms) in 2004/05 and 2008/09 and also had data on CRP on those same occasions, had follow-up data on depression status in 2012/13, and had data on covariates in 2008/09. Compared with participants who did not have depressive symptoms on these two baseline occasions but were excluded from the analytic sample (maximum *n*=2667), those included did not differ by sex (*P*=0.89) but were younger (67.3 vs 70.0 years, *P*<0.001) and less likely to be in the lowest wealth quintile (12.5% vs 17.8%, *P*<0.001). Participants included in the sample had a slightly lower BMI (27.9 vs 28.3 kg/m^2^, *P*=0.02), a lower chronic disease burden in terms of type 2 diabetes (1.1% vs 2.1%, *P*=0.01), cardiovascular disease (2.0% vs 3.6%, *P*=0.001), cancer (3.6% vs 6.2%, *P*<0.001) and rheumatoid arthritis (5.2% vs 6.8%, *P*=0.02), and were less likely to be disabled (18.1% vs 29.4%, *P*<0.001). Included participants had a lower prevalence of low physical activity (13.2% vs 26.1%, *P*<0.001) but had a similar smoking prevalence (9.3% vs 11.0%, *P*=0.07), and were less likely to use antidepressant medication in 2012/13 (6.6% vs 9.5%, *P*=0.002). Included participants were less likely to be inflamed on one occasion (22.3% vs 26.6%, *P*=0.001) but were more likely to be inflamed on two occasions (19.7% vs 7.3%, *P*<0.001). The proportion of participants who reported in 2003/04, before completing the baseline assessment, having ever been previously diagnosed with depression by a physician was similar between those included and excluded from the analytic sample (4.7% vs 3.7%, respectively, *P*=0.08).

### Participant characteristics

Characteristics at baseline are shown in Table 1 for those participants who were initially without depressive symptoms in 2004/05 and 2008/09. Of these 2068 participants, 1200 were not inflamed on either of two baseline occasions while 461 were inflamed on one occasion and 407 were inflamed on two occasions. Participants inflamed on two occasions were most likely to be within the lowest wealth quintile (17.2% vs 9.6% for zero occasion, *P*<0.001), had the highest mean BMI (31.1 vs 26.7 kg/m^2^ for zero occasion, *P*<0.001), had the highest rate of current smoking (14.3% vs 7.7% for zero occasions, *P*<0.001) and had the highest rate of low physical activity (20.4% vs 9.3%, *P*<0.001). Participants inflamed on two occasions were also most likely to be disabled (25.8% vs 14.7% for zero occasions, *P*<0.001) and had the highest frequency of chronic diseases except for type 2 diabetes, cardiovascular disease and cancer. The distribution of CRP at each measurement occasion was positively skewed with a median (range) of 1.70 (0.20, 210.0) in 2004/05 and of 1.80 (0.20, 126.0) in 2008/09.

### Number of occasions with systemic inflammation and change in the number of depressive symptoms

We first examined the number of occasions inflamed in relation to continuous change in the number of depressive symptoms. Change scores for the number of depressive symptoms from 2008/09 to 2012/13 were normally distributed around 0 with a small positive tendency (mean=0.30, s.d.=1.42). In models adjusted for age, sex and wealth, 1 vs 0 occasion of inflammation was not associated with a mean increase in depressive symptoms (−0.05, 95% confidence intervals (CIs)=−0.21, 0.10, symptoms), while 2 vs 0 occasions of inflammation were associated with a small increase (0.19, 95% CI=0.03, 0.35 symptoms). This increase was modest without statistical significance after adjusting for all measured covariates (0.10, 95% CI=−0.07, 0.28 symptoms). No strong evidence was observed for an interaction between the number of occasions inflamed and participant sex in relation to continuous change in the number of depressive symptoms (*P*=0.18).

### Number of occasions with systemic inflammation and odds of developing depressive symptomology

One hundred and thirty-one new cases of depressive symptomology (more than or equal to four of eight symptoms) were observed in 2012/13. The crude rate of developing depressive symptoms was similar among adults inflamed on 1 vs 0 occasions (5.9% vs 5.2%, *P*=0.58) but increased among those inflamed on 2 vs 0 occasions (10.3% vs 5.2%, *P*<0.001); Table 1). Adjusting for age, sex and wealth (Table 2), adults who were inflamed on 1 vs 0 occasion showed no higher odds of developing depressive symptoms, while those inflamed on 2 vs 0 occasions showed 1.85 (95% CI=1.22, 2.80) times higher odds. Excess risk among those inflamed on two occasions remained elevated after alternative adjustments for health and behavioural covariates and for all measured covariates (odds ratio (OR)=1.60, 95% CI=1.00, 2.55 vs 0 occasions, *P*=0.05).

Some evidence for an interaction was found between the number of occasions inflamed and participant sex in relation to odds of developing depressive symptoms (*P*-interaction=0.035). In sex-stratified analyses adjusting for age and wealth (Table 3), men who were inflamed on 2 vs 0 occasions did not have increased odds of developing depressive symptoms (OR=0.98, 95% CI=0.45, 2.15), while women who were inflamed on 2 vs 0 occasions showed markedly increased odds (OR=2.61, 95% CI=1.56, 4.39); these remained elevated after adjustment for all measured covariates (OR=2.75, 95% CI=1.53, 4.95).

### Number of occasions with systemic inflammation and odds of developing individual depressive symptoms

**Figure 2:**
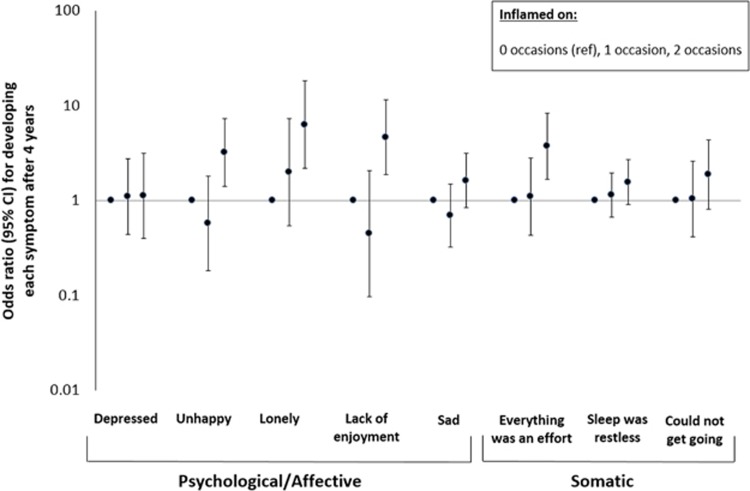
Odds of developing individual depressive symptoms after 4 years based on the number of occasions inflamed (*n*=813). Symptoms refer to experiences in the week preceding assessment. Sample includes 497 participants inflamed on zero occasions, 178 participants inflamed on one occasion and 138 participants inflamed on two occasions.

When examining the number of occasions inflamed in relation to odds of developing each depressive symptom in 2012/13 among a subset of participants initially free of all symptoms in both 2004/05 and 2008/09 (*n*=813, Figure 2), associations were observed only for 2 vs 0 occasions of inflammation with one ‘somatic’ depressive symptom, feeling that everything was an effort (OR=3.73, 95% CI=1.66, 8.35 vs 0 occasions) and three ‘psychological’ depressive symptoms, unhappiness (OR=3.22, 95% CI=1.41, 7.36 vs 0 occasions), loneliness (OR=6.33, 95% CI=2.18, 18.35 vs 0 occasions) and lack of enjoyment (OR=4.67, 95% CI=1.90, 11.52 vs 0 occasions). Evidence was weaker for an association of 2 vs 0 occasions of inflammation with symptoms of sadness (OR=1.63, 95% CI=0.85, 3.14), restless sleep (OR=1.57, 95% CI=0.92, 2.70), difficulty getting going (OR=1.89, 0.81, 4.37); evidence was weakest for the symptom of depression (OR=1.12, 95% CI=0.40, 3.13).

### Sensitivity analyses

To examine whether excluding participants with acute inflammation modified our results, we further excluded participants with a CRP value ⩾10 mg l^−1^. Similar to previous results, increased risk of developing depressive symptoms was observed among those with 2 vs 0 occasions of inflammation (OR=1.73, 95% CI=1.04, 2.90 with adjustment for all measured covariates; Supplementary Appendix 1). In sex-stratified analyses (Supplementary Appendix 2), 2 vs 0 occasions of inflammation were associated with 3.41 (95% CI=1.81, 6.40) times higher odds of developing depressive symptoms among women. This association was not observed in men.

## Discussion

This study aimed to determine whether repeated exposure to systemic inflammation increases the risk of developing new depressive symptoms. Within a population-based sample of older adults who were initially free of depressive symptoms based on having fewer than four of eight symptoms on two prior occasions, our results suggested that being inflamed on both of vs neither of these same occasions conferred an increased risk of future depressive symptomology. The same finding was observed whether analysing depressive symptoms as continuous change or considering positive cases as having at least four of eight symptoms. This association appeared to be sex-specific such that repeated inflammation was associated with increased risk among women, but not among men. Findings of sex-interaction were sensitive to the modelling strategy suggesting that associations either reflect subgroup differences or artefacts of inherent biases.

We first examined worsened depressive symptom status by relating continuous change in the number of depressive symptoms to the number of occasions inflamed, among participants initially free of depressive symptomology; these results suggested an association for repeated inflammation that was small in magnitude, possibly reflecting the rarity of change in depressive symptoms in this non-clinical sample or non-linear outcome associations. We then examined worsened symptom status through the likelihood of having at least four of eight depressive symptoms as done previously.^26, 27, 28^ These results suggested that multiple occasions of inflammation increased risk of future depressive symptoms among women only, an association that was robust to adjustment for a range of social, health and behavioural factors and which persisted after excluding women with very high CRP levels, together suggesting that excess risk did not generally reflect a higher burden of chronic disease or occult infection. Our results agree with one recent study, which reported that high levels of IL-6 measured on two separate occasions had greater predictive power for future depressive symptoms than high IL-6 measured on a single occasion,^33^ suggesting that inflammation confers greater risk of new depressive symptoms when exposure persists over time. CRP is a relatively large protein thought to be less responsive to minor fluctuations in health than small cytokines such as IL-6,^34^ and repeated measures of CRP may therefore be useful for indicating long-term exposure to inflammation; this has not been previously examined.

Furthermore, given that somatic symptoms of depression may reflect adaptive energy-conserving responses to inflammation^17^ these symptoms may be expected to develop most readily in response to repeated inflammation; a theory supported by recent cross-sectional results showing only depressive symptoms of a somatic nature to be associated with raised CRP.^35^ This symptom pattern was not clearly observed in the present study, with repeated inflammation predicting development of three symptoms that may be deemed psychological or affective (unhappiness, loneliness and lack of enjoyment) and only one symptom that may be deemed somatic (feeling everything was an effort); this may however reflect low statistical power, given positive effect sizes for other somatic symptoms with trends towards significance. The older age of participants in this study may have also influenced the type of symptoms that were associated with inflammation, given that these may be conditioned by declining physical health, multi-morbidity and loss of social networks;^36^ symptoms may differ from those arising in earlier life stages. Data on early life symptoms were not available and so it was not possible to examine this issue or indeed determine whether symptoms observed here were truly new or a recurrence of previous symptomology.

Systemic inflammation may exert depressogenic effects through several mechanisms including disrupting the metabolism of the neurotransmitters serotonin and dopamine,^37^ impairing reward sensitivity in the basal ganglia and threat sensitivity in the anterior cingulate cortex,^5^ hyper-activation of the hypothalamic–pituitary–adrenal axis,^6, 38^ and inducing glucocorticoid resistance.^4^ Such pathways may be triggered by persistent inflammation, but it is not yet clear why such mechanisms would have stronger effects on mood states among women. Most of the previous studies that reported associations between peripheral inflammation and new depressive symptoms did not examine sex differences, and the one study that did so did not find strong evidence for differential associations between inflammation and depression among men and women.^8^ None of the previous studies which found no effect of inflammation on future depressive symptoms seemed to examine sex differences.^12, 13, 14^ Twice as many new cases of depressive symptoms were observed among women than men in the present study, which could reflect genuine sex differences in inflammatory reactivity to stressful stimuli,^15^ in depressive effects of inflammatory cytokines,^19^ or in wider adaptations related to pathogen–host defence.^17^ One recent study found higher total white blood cell count to predict an increase in depressive symptoms over time among women only^39^ providing further evidence for sex-specificity; risk was not examined in relation to persistency of inflammation. Alternatively, differential associations by sex may reflect methodological issues, such as inferior measurement of depressive symptoms among men, artefacts of selection bias or statistical false positives.

Mood-altering effects of inflammation have some support from trial evidence on clinical populations, which suggests that administration of the pro-inflammatory cytokine interferon-alpha induces depressive symptoms among previously non-depressed adults,^40^ and that administration of an anti-inflammatory TNF-α antagonist reduces depressive symptoms in patients with high CRP levels.^41^ However, a recent population-based study using Mendelian randomisation analyses found no evidence of association between genetically elevated CRP and depressive symptoms,^42^ indicating that circulating CRP itself may not be a causal factor within the wider population. As a marker of inflammation measured in the periphery, high CRP may reflect activity of more upstream pro-inflammatory factors, such as IL-6 and IL-1B which may themselves induce changes in brain function predisposing to depression.^4, 43^

### Strengths and limitations

Strengths of this study include its use of repeated measures on a well-validated marker of systemic inflammation in order to examine the issue of persistent versus transient exposure, a population-based sample, and a prospective design with 4 years of follow-up. We used a self-reported symptom scale to define depression-related phenomena that allows not for a clinical diagnosis but an assessment of subjective mood. Study limitations include its observational design that limits causal inference, given the possibility of residual confounding and reverse causation bias. Data were absent on use of anti-inflammatory or related prescription drugs at baseline that may have affected levels of systemic inflammation; rates of prescription drug use are however expected to be high throughout this population, given their older age and are not likely specific to those who are systemically inflamed. Alcohol consumption was not included as a covariate due to the volume consumed not being measured and due to a large degree of missing data on other aspects of consumption, which would have further reduced the precision of results. Consumption of alcohol and a range of food groups have been associated positively with systemic inflammation^44^ and may subsequently lead to depressive symptoms, but high alcohol consumption is also known to cluster with other health behaviours including smoking and low physical activity,^45^ both of which were considered here. Participants were of an older age when exclusion of those with depressive symptoms at baseline were made, which may have missed severe cases or symptoms that developed earlier in life. Participants analysed were also relatively healthy compared with the source population; this could result in conservative effect estimates but also in artefacts of selection bias. The prospective study design restricted the number of participants available for analyses and resulted in some imprecision in effect estimates by way of relatively wide confidence intervals indicating that effect sizes should be interpreted with caution.

## Conclusions

In this cohort study of older adults, repeated, but not transient, exposure to systemic inflammation was associated with increased risk of future depressive symptoms among women. This subgroup finding requires causal analyses spanning other age groups to confirm its validity. Intervention studies would ultimately be needed to determine whether reducing systemic inflammation through use of anti-inflammatory drugs or lifestyle interventions would prevent the onset of depressive symptoms.

## Author contributions

JAB had full access to the data and takes primary responsibility for the integrity and accuracy of results. LAC obtained funding, supervised, contributed to the concept and design of study, drafting and all authors contributed to the critical revision of the manuscript.

## Figures and Tables

**Table 1:** Baseline characteristics of adults without depression according to the number of occasions inflamed over the observation period (*n*=2068)

	*Inflamed on zero occasions (*n*=1200)*	*Inflamed on one occasion (*n*=461)*	*Inflamed on two occasions (*n*=407)*
*Baseline characteristics (2008/09)*			
Age—mean (s.d.)	66.7 (7.6)	68.6 (8.3)^a^	67.5 (7.9)
Female	612 (51.0)	236 (51.2)	242 (59.5)^a^
Lowest wealth quintile	115 (9.6)	73 (15.8)^a^	70 (17.2)^a^
Uses antidepressant medication	68 (5.7)	28 (6.1)	41 (10.1)^a^
Body mass index—mean (s.d.)	26.7 (3.9)	28.5 (4.6)^a^	31.1 (5.7)^a^
Reported type 2 diabetes	14 (1.2)	4 (0.9)	5 (1.2)
Reported cardiovascular disease	19 (1.6)	15 (3.3)^a^	7 (1.7)
Reported cancer	38 (3.2)	23 (5.0)	14 (3.4)
Reported osteoarthritis	270 (22.5)	110 (23.9)	118 (29.0)^a^
Reported rheumatoid arthritis	52 (4.3)	21 (4.6)	34 (8.4)^a^
Reported chronic lung disease	27 (2.3)	18 (3.9)	26 (6.4)^a^
Reported asthma	94 (7.8)	54 (11.7)^a^	58 (14.3)^a^
Disabled based on activity limitations	176 (14.7)	93 (20.2)^a^	105 (25.8)^a^
Cognitively impaired	135 (11.3)	78 (16.9)^a^	72 (17.7)^a^
Currently smokes	92 (7.7)	43 (9.3)	58 (14.3)^a^
Low physical activity	111 (9.3)	79 (17.1)^a^	83 (20.4)^a^
New depressive symptoms after 4 years^b^	62 (5.2)	27 (5.9)	42 (10.3)^a^

Data are *n* (%) unless otherwise noted.

a Indicates significantly different from the ‘inflamed on zero occasions’ group (*P*<0.05) based on linear or logistic regression.

b Measured at follow-up (2012/13).

**Table 2:** Odds of developing depressive symptoms after 4 years among adults initially without depressive symptoms, based on the number of occasions inflamed (*n*=2068)

	*Odds of developing depressive symptoms after 4 years*				
	*Model 1 (age, sex and wealth)*	*Model 1+antidepressant drug use*^a^	*Model 1+BMI, chronic disease*^b^*, disability and cognitive impairment*	*Model 1+smoking and physical activity*	*Adjusted for all factors*
	*Odds ratio (95% CI)*	*Odds ratio (95% CI)*	*Odds ratio (95% CI)*	*Odds ratio (95% CI)*	*Odds ratio (95% CI)*
*Inflammatory status*					
Inflamed on zero occasions (*n*=1200)	1.00 (reference)	1.00 (reference)	1.00 (reference)	1.00 (reference)	1.00 (reference)
Inflamed on one occasion (*n*=461)	1.03 (0.64, 1.65)	1.03 (0.64, 1.65)	0.98 (0.61, 1.60)	0.98 (0.61, 1.58)	0.96 (0.59, 1.56)
Inflamed on two occasions (*n*=407)	1.85 (1.22, 2.80)	1.78 (1.17, 2.71)	1.74 (1.10, 2.75)	1.68 (1.10, 2.57)	1.60 (1.00, 2.55)

Abbreviations: CI, confidence interval; CRP, C-reactive protein. The number of occasions inflamed considers having CRP ⩾3 mg l^−1^ at either none of, one of or both of 2004/05 and 2008/09. Outcome defined as having depressive symptoms (more than or equal to four of eight symptoms) vs not having depressive symptoms (less than four of eight symptoms) in 2012/13. Covariates are assessed in 2008/09.

a Antidepressant drug use based on nurse-coded drugs in 2012/13.

b Chronic disease considers prevalent/recent cardiovascular disease (myocardial infarction, angina or stroke), type 2 diabetes, cancer, osteoarthritis, rheumatoid arthritis, chronic lung disease and asthma.

**Table 3:** Odds of developing depressive symptoms after 4 years among men and women initially without depressive symptoms, based on the number of occasions inflamed (*n*=2068)

	*Odds of developing depressive symptoms after 4 years*				
	*Model 1 (age and wealth)*	*Model 1+antidepressant drug use*^a^	*Model 1+BMI, chronic disease*^b^*, disability and cognitive impairment*	*Model 1+smoking and physical activity*	*Adjusted for all factors*
	*Odds ratio (95% CI)*	*Odds ratio (95% CI)*	*Odds ratio (95% CI)*	*Odds ratio (95% CI)*	*Odds ratio (95% CI)*
*Among men*					
Inflamed on zero occasions (*n*=588)	1.00 (reference)	1.00 (reference)	1.00 (reference)	1.00 (reference)	1.00 (reference)
Inflamed on one occasion (*n*=225)	0.44 (0.18, 1.08)	0.44 (0.18, 1.09)	0.40 (0.16, 1.02)	0.40 (0.16, 1.00)	0.38 (0.15, 0.98)
Inflamed on two occasions (*n*=165)	0.98 (0.45, 2.15)	0.96 (0.44, 2.12)	0.79 (0.34, 1.82)	0.83 (0.36, 1.87)	0.70 (0.29, 1.68)
					
*Among women*					
Inflamed on zero occasions (*n*=612)	1.00 (reference)	1.00 (reference)	1.00 (reference)	1.00 (reference)	1.00 (reference)
Inflamed on one occasion (*n*=236)	1.63 (0.91, 2.90)	1.62 (0.91, 2.89)	1.66 (0.91, 3.01)	1.62 (0.90, 2.92)	1.67 (0.91, 3.06)
Inflamed on two occasions (*n*=242)	2.61 (1.56, 4.39)	2.51 (1.49, 4.22)	2.83 (1.58, 5.04)	2.55 (1.50, 4.32)	2.75 (1.53, 4.95)

Abbreviations: CI, confidence interval; CRP, C-reactive protein. The number of occasions inflamed considers having CRP ⩾3 mg l^−1^ at either none of, one of or both of 2004/05 and 2008/09. Outcome defined as having depressive symptoms (more than or equal to four of eight symptoms) vs not having depressive symptoms (less than four of eight symptoms) in 2012/13. Covariates are assessed in 2008/09.

a Antidepressant drug use based on nurse-coded drugs in 2012/13.

b Chronic disease considers prevalent/recent cardiovascular disease (myocardial infarction, angina or stroke), type 2 diabetes, cancer, osteoarthritis, rheumatoid arthritis, chronic lung disease and asthma.
