# Supplementary material for: Repeated exposure to systemic inflammation and risk of new depressive symptoms among older adults
Source: Transl Psychiatry. 2017 Aug 15;7(8):e1208–. doi: 10.1038/tp.2017.155 (PMC5611724; doi:10.1038/tp.2017.155)
Supplement: Supplementary Appendix [file tp2017155x1.docx]

| **Appendix 1** Odds of depressed mood after 4 years among adults initially without depressed mood, based on the number of occasions inflamed, excluding individuals with CRP ≥ 10 mg/l on each occasion (n=1887) | | | | | | |
| --- | --- | --- | --- | --- | --- | --- |
|  | | | | | | |
|  |  | **Odds of developing depressed mood after 4 years** | | | | |
|  |  |  |  |  |  |  |
|  |  | **Model 1**  **Age, sex, wealth** | **Model 1 +**  **Antidepressant**  **drug use^1^** | **Model 1 +**  **BMI, chronic disease^2^, disability, cognitive impairment** | **Model 1 +**  **Smoking,**  **physical activity** | **Adjusted**  **for all factors** |
|  |  | Odds Ratio (95% CI) | Odds Ratio (95% CI) | Odds Ratio (95% CI) | Odds Ratio (95% CI) | Odds Ratio (95% CI) |
| ***Inflammatory status*** |  |  |  |  |  |  |
| Inflamed on 0 occasions (n=1200) |  | 1.00 (reference) | 1.00 (reference) | 1.00 (reference) | 1.00 (reference) | 1.00 (reference) |
| Inflamed on 1 occasion (n=400) |  | 0.92 (0.55, 1.55) | 0.92 (0.55, 1.54) | 0.88 (0.52, 1.49) | 0.89 (0.53, 1.50) | 0.85 (0.50, 1.45) |
| Inflamed on 2 occasions (n=287) |  | 1.90 (1.20, 3.03) | 1.83 (1.15, 2.93) | 1.84 (1.11, 3.05) | 1.80 (1.13, 2.89) | 1.73 (1.04, 2.90) |
|  |  |  |  |  |  |  |
| Number of occasions inflamed considers having CRP ≥ 3 mg/L at either none of, 1 of, or both of 2004/05 and 2008/09. Outcome defined as having depressed mood vs. not in 2012/13. Covariates are assessed in 2008/09. ^1^Antidepressant drug use based on nurse-coded drugs in 2012/13. ^2^Chronic disease considers prevalent/recent cardiovascular disease (myocardial infarction, angina, or stroke), type 2 diabetes, cancer, osteoarthritis, rheumatoid arthritis, chronic lung disease, and asthma. | | | | | | |

| **Appendix 2** Odds of depressed mood after 4 years among men and women initially without depressed mood, based on the number of occasions inflamed, excluding individuals with CRP ≥ 10 mg/l on each occasion (n=1887) | | | | | | |
| --- | --- | --- | --- | --- | --- | --- |
|  | | | | | | |
|  |  | **Odds of developing depressed mood after 4 years** | | | | |
|  |  |  |  |  |  |  |
|  |  | **Model 1**  **Age, wealth** | **Model 1 +**  **Antidepressant**  **drug use^1^** | **Model 1 +**  **BMI, chronic disease^2^, disability, cognitive impairment** | **Model 1 +**  **Smoking,**  **physical activity** | **Adjusted**  **for all factors** |
|  |  | Odds Ratio (95% CI) | Odds Ratio (95% CI) | Odds Ratio (95% CI) | Odds Ratio (95% CI) | Odds Ratio (95% CI) |
| ***Among men*** |  |  |  |  |  |  |
| Inflamed on 0 occasions (n=588) |  | 1.00 (reference) | 1.00 (reference) | 1.00 (reference) | 1.00 (reference) | 1.00 (reference) |
| Inflamed on 1 occasion (n=191) |  | 0.25 (0.08, 0.85) | 0.26 (0.08, 0.86) | 0.22 (0.06, 0.76) | 0.23 (0.07, 0.79) | 0.20 (0.06, 0.72) |
| Inflamed on 2 occasions (n=119) |  | 0.74 (0.28, 1.99) | 0.72 (0.27, 1.94) | 0.58 (0.20, 1.67) | 0.65 (0.23, 1.79) | 0.52 (0.17, 1.56) |
|  |  |  |  |  |  |  |
| ***Among women*** |  |  |  |  |  |  |
| Inflamed on 0 occasions (n=612) |  | 1.00 (reference) | 1.00 (reference) | 1.00 (reference) | 1.00 (reference) | 1.00 (reference) |
| Inflamed on 1 occasion (n=209) |  | 1.61 (0.87, 2.95) | 1.59 (0.87, 2.92) | 1.69 (0.90, 3.16) | 1.64 (0.88, 3.04) | 1.70 (0.90, 3.22) |
| Inflamed on 2 occasions (n=168) |  | 2.98 (1.69, 5.23) | 2.87 (1.63, 5.06) | 3.40 (1.83, 6.32) | 3.09 (1.74, 5.49) | 3.41 (1.81, 6.40) |
|  |  |  |  |  |  |  |
| Number of occasions inflamed considers having CRP ≥ 3 mg/L at either none of, 1 of, or both of 2004/05 and 2008/09. Outcome defined as having depressed mood vs. not in 2012/13. Covariates are assessed in 2008/09. ^1^Antidepressant drug use based on nurse-coded drugs in 2012/13. ^2^Chronic disease considers prevalent/recent cardiovascular disease (myocardial infarction, angina, or stroke), type 2 diabetes, cancer, osteoarthritis, rheumatoid arthritis, chronic lung disease, and asthma. | | | | | | |

**Appendix 3**

**MRC ImmunoPsychiatry Consortium**

**University of Cambridge**

Edward Bullmore ([etb23@medschl.cam.ac.uk](mailto:etb23@medschl.cam.ac.uk))

Petra E. Vértes ([pv226@cam.ac.uk](mailto:pv226@cam.ac.uk))

Rudolf Cardinal ([rnc1001@cam.ac.uk](mailto:rnc1001@cam.ac.uk))

*Department of Psychiatry, Behavioural and Clinical Neuroscience Institute, University of Cambridge, Cambridge CB2 0SZ*

**MRC Biostatistics Unit (Cambridge)**

Sylvia Richardson ([sylvia.richardson@mrc-bsu.cam.ac.uk](mailto:sylvia.richardson@mrc-bsu.cam.ac.uk))

Gwenael Leday ([gwenael@mrc-bsu.cam.ac.uk](mailto:gwenael@mrc-bsu.cam.ac.uk))

*MRC Biostatistics Unit, Cambridge Institute of Public Health, Forvie Site, Robinson Way, Cambridge Biomedical Campus, Cambridge CB2 0SR*

**University of Edinburgh**

Tom Freeman ([tfreeman@roslin.ed.ac.uk](mailto:tfreeman@roslin.ed.ac.uk))

David Hume ([david.hume@roslin.ed.ac.uk](mailto:david.hume@roslin.ed.ac.uk))

Tim Regan ([Tim.Regan@roslin.ed.ac.uk](mailto:Tim.Regan@roslin.ed.ac.uk))

Zhaozong Wu ([Zhaozong.Wu@roslin.ed.ac.uk](mailto:Zhaozong.Wu@roslin.ed.ac.uk))

*System Immunology Group, Division of Genetics and Genomics, The Roslin Institute and Royal (Dick) School of Veterinary Studies, University of Edinburgh, Easter Bush, Midlothian EH25 9RG*

**King’s College London**

Carmine Pariante ([carmine.pariante@kcl.ac.uk](mailto:carmine.pariante@kcl.ac.uk))

Annamaria Cattaneo ([annamaria.cattaneo@kcl.ac.uk](mailto:annamaria.cattaneo@kcl.ac.uk))

Patricia Zuszain ([patricia.zunszain@kcl.ac.uk](mailto:patricia.zunszain@kcl.ac.uk))

Alessandra Borsini ([alessandra.borsini@kcl.ac.uk](mailto:alessandra.borsini@kcl.ac.uk) )

*Psychiatry and Immunology Lab & Perinatal Psychiatry, Institute of Psychiatry, Psychology and Neuroscience, King’s College London G.32.01, The Maurice Wohl Clinical Neuroscience Institute, Cutcombe Road, London SE5 8AF*

Robert Stewart ([robert.stewart@kcl.ac.uk](mailto:robert.stewart@kcl.ac.uk))

David Chandran ([david.chandran@kcl.ac.uk](mailto:david.chandran@kcl.ac.uk))

*Institute of Psychiatry, Psychology and Neuroscience, King’s College London, The Maurice Wohl Clinical Neuroscience Institute, Cutcombe Road, London SE5 9RT*

**Queen Mary University of London (previously at UCL)**

Livia Carvalho ([l.carvalho@qmul.ac.uk](mailto:l.carvalho@qmul.ac.uk) )

Joshua Bell ([joshua.bell.11@ucl.ac.uk](mailto:joshua.bell.11@ucl.ac.uk))

Luis Henrique Souza-Teodoro ([lhteodoro@gmail.com](mailto:lhteodoro@gmail.com))

Department of Clinical Pharmacology, William Harvey Research Institute, Charterhouse Square, Barts and the London School of Medicine and Dentistry, Queen Mary University of London, EC1M 6BQ

**University of Southampton**

Hugh Perry ([V.H.Perry@soton.ac.uk](mailto:V.H.Perry@soton.ac.uk))

*Centre for Biological Sciences, University of Southampton, Mail Point 840 LD80B, South Lab and Path Block, Southampton General Hospital, SO16 6YD*

**University of Sussex**

Neil Harrison ([N.Harrison@bsms.ac.uk](mailto:N.Harrison@bsms.ac.uk))

*Brighton & Sussex Medical School, University of Sussex, Brighton BN1 9RR, United Kingdom*

**Janssen**

Wayne Drevets ([wdrevets@ITS.JNJ.com](mailto:wdrevets@ITS.JNJ.com)

*Janssen Pharmaceuticals of Johnson and Johnson, Janssen Research & Development, 1125 Trenton-Harbourton Road, Titusville, NJ 08560*

Gayle M Wittenberg ([GWittenb@ITS.JNJ.com](mailto:GWittenb@ITS.JNJ.com))

Yu Sun ([YSun25@its.jnj.com](mailto:YSun25@its.jnj.com))

*Janssen Research & Development, 1125 Trenton-Harbourton Road, Titusville, NJ 08560*

Declan Jones ([djone119@ITS.JNJ.com](mailto:djone119@ITS.JNJ.com))

*J&J Innovation Centre, One Chapel Place London W1G 0BG*

**GlaxoSmithKline**

Edward Bullmore ([Edward.t.bullmore@gsk.com](mailto:Edward.t.bullmore@gsk.com))

*GSK Clinical Unit in Cambridge Building, Addenbrookes Hospital, Hills Road, Cambridge CB2 2GG*

Shahid Khan ([shahid.a.khan@gsk.com](mailto:shahid.a.khan@gsk.com))

Annie Stylianou ([anastasia.2.Stylianou@gsk.com](mailto:anastasia.2.Stylianou@gsk.com))

Robert B Henderson ([Robbie.b.henderson@gsk.com](mailto:Robbie.b.henderson@gsk.com))

*GSK, 1 Gunnelswood Road, Stevenage SG1 2NY*
